# 3D-Printed Soft Membrane for Periodontal Guided Tissue Regeneration

**DOI:** 10.3390/ma16041364

**Published:** 2023-02-06

**Authors:** Farshid Vahdatinia, Amirarsalan Hooshyarfard, Shokoofeh Jamshidi, Setareh Shojaei, Kishan Patel, Erfan Moeinifard, Rasool Haddadi, Maryam Farhadian, Leila Gholami, Lobat Tayebi

**Affiliations:** 1 Dental Implants Research Center, Hamadan University of Medical Sciences, Hamadan 6517838636, Iran; 2 Department of Periodontics, Faculty of Dentistry, Tehran Medical Sciences, Islamic Azad University, Tehran 1946853314, Iran; 3 Department of Oral and Maxillofacial Pathology, School of Dentistry, Dental Research Center, Hamadan University of Medical Sciences, Hamadan 6517838636, Iran; 4 Department of Oral and Maxillofacial Pathology, School of Dentistry, Hamadan University of Medical Sciences, Hamadan 6517838636, Iran; 5 School of Dentistry, Marquette University, Milwaukee, WI 53233, USA; 6 Royal Veterinary Clinic, Hamadan 6516835632, Iran; 7 Department of Pharmacology and Toxicology, School of Pharmacy, Hamadan University of Medical Sciences, Hamadan 6517838636, Iran; 8 Department of Biostatistics, School of Public Health, Research Center for Health Sciences, Hamadan University of Medical Sciences, Hamadan 6517838636, Iran; 9 Dental Research Center, Hamadan University of Medical Sciences, Hamadan 6517838636, Iran

**Keywords:** periodontal regeneration, tissue engineering, 3D-printed membrane, guided tissue regeneration (GTR), periodontal, membranes

## Abstract

Objectives: The current study aimed to perform an in vivo examination using a critical-size periodontal canine model to investigate the capability of a 3D-printed soft membrane for guided tissue regeneration (GTR). This membrane is made of a specific composition of gelatin, elastin, and sodium hyaluronate that was fine-tuned and fully characterized in vitro in our previous study. The value of this composition is its potential to be employed as a suitable replacement for collagen, which is the main component of conventional GTR membranes, to overcome the cost issue with collagen. Methods: Critical-size dehiscence defects were surgically created on the buccal surface of the roots of canine bilateral mandibular teeth. GTR treatment was performed with the 3D-printed membrane and two commercially available collagen membranes (Botiss Jason^®^ and Smartbrane-Regedent membranes) and a group without any membrane placement was considered as the control group. The defects were submerged with tension-free closure of the gingival flaps. Histologic and histometric analyses were employed to assess the periodontal healing over an 8-week experimental period. Results: Histometric evaluations confirmed higher levels of new bone formation in the 3D-printed membrane group. Moreover, in all defects treated with the membranes, the formation of periodontal tissues, bone, periodontal ligaments, and cementum was observed after 8 weeks, while in the control group, only connective tissue was found in the defect sites. There was no clinical sign of inflammation or recession of gingiva in any of the groups. Significance: The 3D-printed gelatin/elastin/sodium hyaluronate membrane can be safe and effective for use in GTR for periodontal tissue regeneration therapies, with better or comparable results to the commercial collagen membranes.

## 1. Introduction

Untreated chronic periodontitis results in the destruction of tooth-supporting structures of the gingiva, alveolar bone, periodontal ligaments, and cementum, and may eventually lead to tooth loss. Although periodontitis can be controlled with the management of bacterial plaque accumulation and the resulting inflammatory reaction, regeneration of the lost periodontal tissues is still a challenge in periodontology. Numerous invasive and non-invasive methods have been introduced with the aim of repairing damaged periodontal tissues and restoring their original form and function [1,2].

Guided bone/tissue regeneration (GBR/GTR) techniques are surgical techniques used to regenerate lost bone/tissue and are accepted treatment modalities for alveolar bone augmentation and the reconstruction of lost periodontal structures in periodontal defects. Barrier membranes are used in these techniques to impede the ingrowth of soft tissue into defect sites and to promote periodontal tissue or bone regeneration [3,4,5]. Basically, a GTR/GBR membrane is a type of medical device that is used to separate and protect the regenerating bone from surrounding tissues. They are often used in dental implant site development to restore the alveolar bone lost due to tooth extraction, when they are referred to as GBR, or in procedures that aim to regenerate the whole periodontal structure lost around a tooth root, when they are regarded as GTR.

According to Melcher’s theory, the basis of the GTR technique is the placement of an occluding material between the gingival tissue and the bone defect space to prevent the rapid transfer of epithelial tissue and connective tissue into the wound space, as well as to assist in bone, cementum, and periodontal ligament replacement [6,7,8]. Many different generations of membranes have been developed for this purpose that share basic properties of space maintenance, tissue integration, biocompatibility, cell conclusiveness, and good surgical manageability [9,10,11,12].

Some features of membranes that are still being modified in different designs are the size of the pores, biodegradability, and mechanical properties. The pore size of a GTR/GBR membrane must prevent the penetration of keratinocyte cells into the defective space but must also allow the vascularization and formation of new bone tissue. The degradation time should be enough to allow complete bone and tissue regeneration. Good mechanical strength and tear resistance are other desirable membrane characteristics that need to be tested and adjusted during the fabrication of membranes for GBR/GTR techniques [13,14]. 

Membranes are divided into two major types: resorbable (degradable) and non-resorbable (non-degradable). Polytetrafluoroethylene (e-PTFE) and titanium mesh (Ti) are two examples of non-resorbable membranes, and collagen is the most important clinically used resorbable membrane [15,16,17]. The major dilemma for non-resorbable membranes is the need for a second surgery for their removal. Therefore, degradable membranes, such as collagen membranes, were introduced to eliminate this disadvantage. The most important features of collagen membranes are their low immune response, low toxicity, ability to stimulate growth and cellular attachment, homeostasis, and ability to regenerate microfibrillar structures [18]. 

Collagen is the basis of the structural stability of many tissues and the most common protein found in bone. Collagen has a homeostatic function to allow early wound stabilization, attracts fibroblasts with its chemotactic properties, and is semi-permeable, which allows for the transmission of nutrients [19]. Although many good clinical results have been achieved through use of collagen membranes, the expense; high absorption rate, especially before the formation of the desired tissue; and poor mechanical properties, which may lead to difficult surgical handling, are disadvantages of this type of membrane [9,12].

Investigating new resorbable membranes with the ability to improve tissue regeneration with optimal mechanical properties, surgical handling, and even customized production characteristics remains an important challenge [20,21,22]. 

Resorbable membranes can be made of synthetic or natural polymers. Polylactic acid (PLA), polyglycolic acid (PGA), polytrimethylene carbonate (PTMC), and poly (ε-caprolactone) (PCL) have been used for this purpose [23,24,25]. Membranes made of synthetic polymers have capabilities such as a controlled degradation rate, mechanical durability, and the ability to be produced through different methods [26]. However, they do not have good biological properties such as cell attachment and integration with the surrounding tissues. Natural polymers and hydrogels have suitable biological properties and compatibility as important factors for tissue regeneration [27,28,29]. Various types of 3D-printed hydrogels have been developed and studied in recent years. Three-dimensional printing of hydrogel-based bioinks, with their versatile structure and composition mimicking the extracellular matrix of human tissue, produces suitable scaffold materials for tissue engineering applications [30,31]. By blending with other polymers, the mechanical properties of such scaffolds can be enhanced. Moreover, the fabrication of novel composite membranes with controlled drug-release properties provides promising novel applications in biomedicine and periodontal treatment [32,33,34]. Developments in the field of biomaterials are ever-growing, and new materials such as elastic and stretchable polymer hydrogel with conducting properties are among these developments with promising applications in fields like cranial surgery, stretchable electronic skins, and intelligent robotic systems [35,36,37,38,39].

We have recently developed a new barrier membrane using 3D-printing technology composed of three hydrogels: elastin, gelatin, and sodium hyaluronate [10]. Gelatin is a denatured form of collagen which has a biological cell affinity and can enhance osteoblast adhesion, migration, and mineralization like collagen but is much less expensive. Although elastin and hyaluronic acid are similarly costly to collagen, they make up a small portion of the membrane but offer significant beneficial properties. The addition of elastin, which is a component of the extracellular matrix, improves elasticity and long-term stability. In addition, applications of hyaluronic acid can facilitate cell signaling, wound healing, morphogenesis, and matrix organization [40]. 

Optimizing the composition of these three hydrogels based on the rheology analyses, printability, and in vitro assessments, we found 8% *w*/*v* gelatin, 2% *w*/*v* elastin, and 0.5% *w*/*v* sodium hyaluronate the most appropriate composition for a membrane with good physical, mechanical, and biological properties [10,41]. 

The appropriate design of membranes can regulate cell activity during tissue engineering [20,21,42,43,44]. Three-dimensional printed technology materials are deposited layer by layer, where the size and degree of porosity of each layer can be controlled. Thus, 3D printing can provide the ability to make a barrier membrane with different structures and pore sizes on different sides [10]. This configuration is very suitable and effective for use in GTR applications, in which we need to accommodate different cell types on different sides [10]. 

This paper is dedicated to conducting an in vivo study on this membrane to show its pre-clinical performance using a relevant canine model for periodontal regeneration. The present study compares this novel 3D-printed membrane with two conventional collagen membranes available in the market for periodontal GTR applications to evaluate its performance, effectiveness, and surgical handling. 

## 2. Materials and Methods

### 2.1. Fabrication and Characterization of the 3D-Printed Membrane

The substances of the membrane were obtained from the following sources:

Elastin with a molecular weight of 60 KDa (referred to as “Elastin-Soluble No. ES12”) was purchased from Elastin Products Company, Inc. (Owensville, MO, USA).

Sodium hyaluronate with a molecular weight range of 500 KDa to 749 KDa was obtained from Lifecore Biomedical (Chaska, MN, USA).

Gelatin (Type A, derived from porcine skin and of bioreagent grade) was obtained from Sigma-Aldrich (St. Louis, MO, USA).

1-Ethyl-3-(3-dimethylaminopropyl) carbodiimide (EDC) and N-Hydroxysuccinimide (NHS) were purchased from Alfa Aesar (Haverhill, MA, USA). 

The ink was made by mixing gelatin, elastin, and sodium hyaluronate at the concentration (*w*/*v*) of 8% gelatin, 2% elastin, and 0.5% sodium hyaluronate in water, based on the optimized compositions studied in our previously published article [10]. The EnvisionTEC 3D-Bioplotter (ENVISIONTEC, Gladbeck, Germany) was used to 3D print the membrane. The 3D-Bioplotter’s camera was used to capture images of each layer during the printing process. The ink was printed at a material container temperature of 30–32 °C, a platform temperature of 11 °C, a printing pressure of 0.6–1.2 bar, and a speed of 20 mm/s using a 250 µm-diameter needle. Pre- and post-flow delays were set to zero. Each membrane had 6 layers with strand angles of 45°, 135°, 0°, 90°, 0°, and 90° for layers 1 through 6, respectively. The distance between strands was set to 0.6 µm for the first 4 layers and 0.9 µm for the last 2 layers. The ink solidified almost instantly after being dispensed from the needle and coming into contact with the platform due to the ability of the solution to form a solid gel at lower temperatures. The 3D-printed membranes were cross-linked by soaking them in 6 mg/mL EDC and 0.75 mg/mL NHS in 70% ethanol for 0.5–2 h. To remove any leftover cross-linker, the membranes were carefully washed by soaking them in 500 mL of DI water for 1.5 h, with the water being replaced with fresh water every 0.5 h. The prepared membranes were stored in 100% ethanol in a −20 °C freezer to be used after rehydration when needed [10]. The detailed 3D-printed procedure of the membrane and relevant characterizations were reported in our previously published articles [10]. 

**Figure 1:**
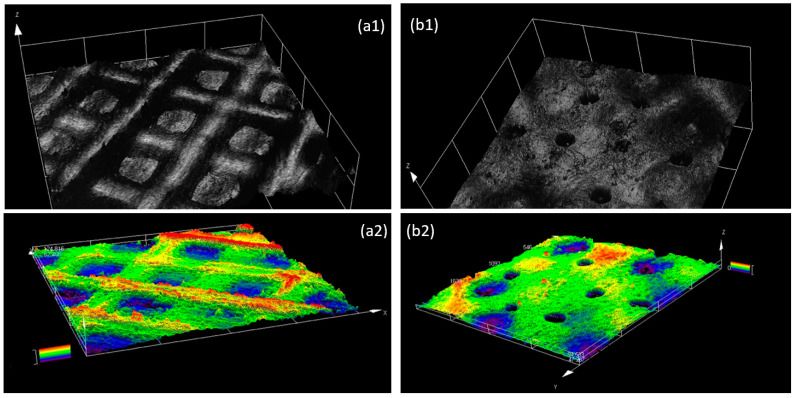
Laser scanning microscopy images of the 3D-printed membrane. Configuration of the surface and height profile of the side with large pores (**a1**,**a2**) and the side with a small pore size (**b1**,**b2**).

A Dino-lite digital microscope camera and a LEXT OLS4000 3D laser measuring microscopy (Olympus, Tokyo, Japan) were used to capture low magnification images and analyze the surface morphology of the membranes after they were prepared (Figure 1). The thickness of the membranes (150 µm) was measured using a Marathon electronic digital micrometer and verified with the 3D laser measuring microscope by scanning the edge of the membrane. 

**Figure 2:**
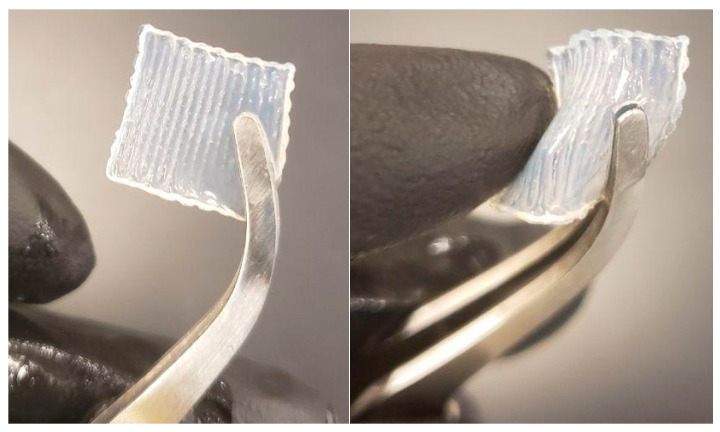
The configuration and flexibility of the 3D-printed membrane allow for its easy surgical handling as confirmed by the surgeon during the in vivo study.

In the membrane designed for GTR application, a membrane including 6 layers with a gradient structure, different pore sizes on different sides, and a total thickness of 150 µm possessed the static tensile modules of 1.95 ± 0.55 MPa and dynamic tensile storage modulus of 314 ± 50 kPa [10]. One side of the membrane had large pores, ranging in size from 400 to 500 µm (2 layers), while the other side had smaller pores, ranging in size from 50 to 150 µm (2 layers). The two layers in the middle of the membrane have a pore size in the range of 200 to 300 150 µm. The in vitro tests showed that the construct can perform the barrier function of a GTR membrane by separating two different cell types (epithelial and connective tissue cells) [10]. The membrane has suitable flexibility to be adapted to the surgical site, and its ease of surgical handling was confirmed by the surgeon during the in vivo study (Figure 2).

### 2.2. Surgical Procedure

Four healthy male canines weighing ~25 kg with a clinically healthy periodontium were used for the present study. The animal selection and management and the surgical procedures were approved by the Ethics Committee on Animal Experimentation (IR.UMSHA.REC.1397.639).

**Figure 3:**
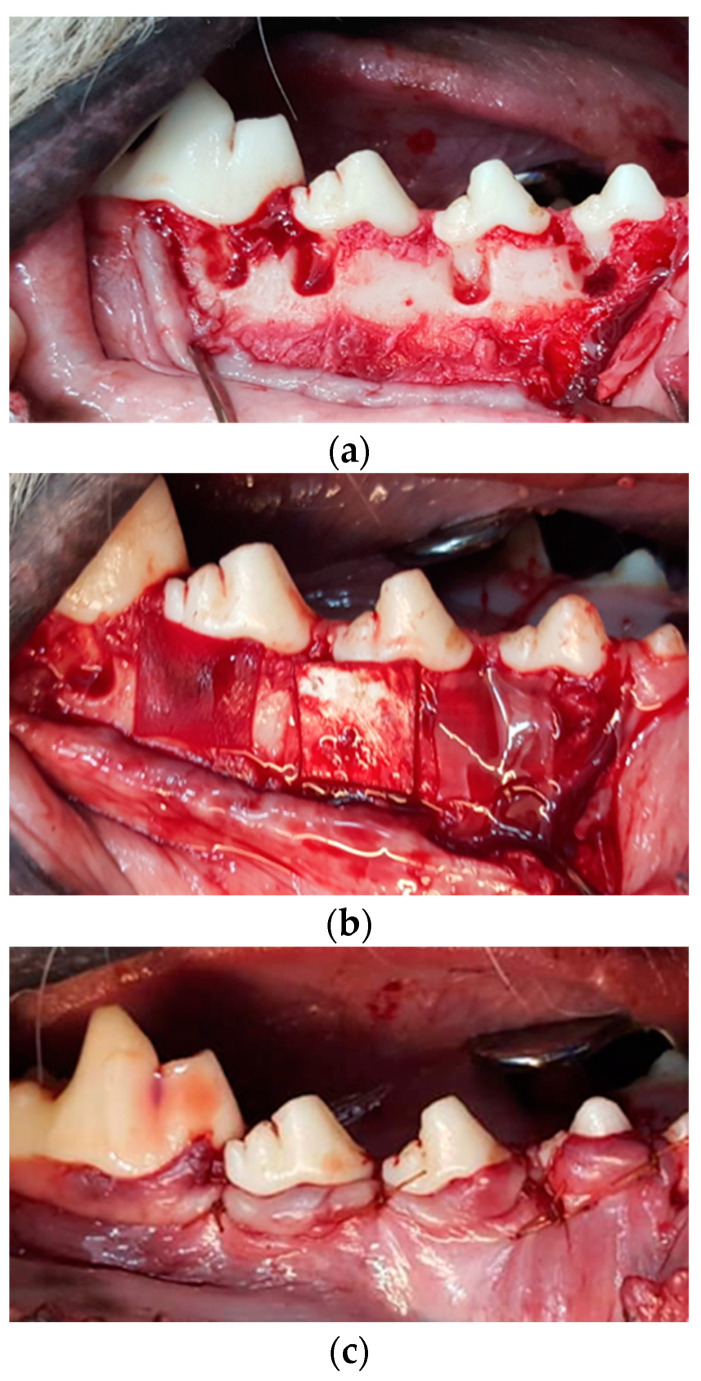
Surgical procedure the preparation of surgically induced dehiscent defects on maxillary premolar teeth. (**a**) The defects (5 × 5 mm below the CEJ) were formed on the buccal side of the distal root of the mandibular premolars and mesial root of first molar teeth, (**b**) Placement of the membranes on defect sites, (**c**) Flap replacement and suturing.

An intramuscular injection of a mixture of Ketamine 10% (Ketamine alfasan, Woerden, Holland, The Netherlands; 10 mg/kg) and Xylazine 2% (5 mg/kg) was used for deep anesthesia. The duration of anesthesia with this method was 1 to 1.5 h. To continue anesthesia, intubation was performed using a combination of oxygen with 1.5% halothane gas (Halothane BP, Nicholas Piramal India Limited, Mumbai, India). Routine dental infiltration anesthesia and lidocaine infiltration (Persocaine-E, Lidocaine HCL 2% + Epinephrine 1/80,000, Daroupakhsh pharmaceutical. Mfg. Co. Tehran, Iran) were used at the surgical sites to control pain and bleeding. Four canines were employed for this study, with a total number of 32 defects. Prior to surgery, oral prophylaxis with 0.2% chlorhexidine was performed. An intra-crevicular incision was made on the buccal aspect of the treated sextants. Following the elevation of the buccal mucoperiosteal flap, four square-shaped dehiscence defects were prepared just below the cemento-enamel junction (CEJ) with dimensions of 5 mm × 5 mm (width × length) on the root surface of the canine, the first and second premolars (distal roots), and the mesial root of the first molars in each side of the mandible (four similar defects in each side of the jaw). The bone defects were prepared using rotating burs under sterile saline irrigation. Root planing was performed using Gracey curettes and chisels, and the cementum and periodontal ligaments were completely removed over the exposed root in the defect area (Figure 3). The CEJ and the most apical portion of the denuded dentin surface were used as histopathological markers to determine the reference points for evaluating bone and periodontal regeneration in the area. 

Each defect was washed with normal saline serum and the 32 defects were randomly assigned to four groups: (1) GTR using Botiss Jason^®^ membrane Botiss Biomaterials GmbH, Zossen Germany (Jason), (2) GTR using Smartbrane membrane, Regedent, Zurich, Switzerland (Smartbrane), (3) The novel 3D-printed membrane, and (4) no membrane (Control) (Figure 3). Each membrane was carefully adapted to the defect to cover 2 mm beyond the defect edges. The placement of the 3D-printed membrane was conducted in a way to have the large pore size (400–500 µm) at the side of the bone tissue and the small pore size (50 to 150 µm) at the side of the soft tissue. This placement was used because the GTR membrane serves to create a physical barrier between the regenerating bone and the surrounding tissue, which helps to prevent the soft tissue from infiltrating the area where the bone is being regenerated and requires a smaller pore size at the side of the soft tissue. Moreover, 400–500 µm is appropriate for the growth of bone tissue. 

The flap was repositioned and sutured tightly using 3-0 ePTFE (Osteogenics Biomedical, Inc., Lubbock, TX, USA) at the cemento-enamel junction. The flaps completely covered the membranes in a tension-free closure. The commercial membranes used in this study were porcine pericardium-derived collagen membranes.

### 2.3. Postsurgical Care

After the surgery, tramadol 50 mg (Tehran Chemie Pharmaceutical Co., Tehran, Iran, 5 mg/kg) and ceftriaxone 1 g (Ceftrax, Jaberebne Haian pharmaceutical Mfg. co, Tehran, Iran) were injected intramuscularly for 7 days. The animals were fed on a soft diet for 14 days, and their mouths were rinsed with 0.2% chlorhexidine daily for plaque control. Sutures were removed under sedation after 14 days. 

### 2.4. Histological Observation and Histomorphometric Assessments

The animals were anesthetized and euthanized at 8 weeks. All animal samples were treated with ketamine and magnesium after 8 weeks of surgery. The sulfate and acepromazine were euthanized, and the experimental teeth and their surrounding soft and hard tissues were removed as a block. The specimens were immediately placed in a 10% formalin solution for 48 h after removal from the mandible. The samples were then rinsed in water for 24 h and immersed in 4% buffered EDTA solution for demineralization. After 20 days, 5-micron histological slices were prepared in the bacco-lingual plane by a microtome (Leica Microsystems, Wetzlar, Germany). They were then stained with Hematoxylin, Eosin, and Goldner trichrome. Finally, a new cement thickness and the greatest thickness of new bone in the defect area were determined by two pathologists unaware of the treatment performed on the specimens.

The prepared sections and slides were photographed by a DP12 U-TV0/5 XC-3 digital camera mounted on an Olympus BX 41 microscope (Olympus, Tokyo, Japan). They were evaluated regarding periodontal healing, new cementum, and bone formation in the defects by a blinded oral pathologist. The thickness and area of the bones were measured using the Analysis LS Starter software(version 5.1, Olympus Soft Imaging Solutions, Tokyo, Japan).

### 2.5. Statistical Analysis

Data (mean ± SD) were analyzed using the nonparametric tests of Kruskal–Wallis and Mann–Whitney. SPSS v.19 software (SPSS INC., Chicago, IL, USA) was used for data analysis. *p* < 0.05 was considered as the level of significance.

## 3. Results

Figure 1 presents the images of a 3D-printed membrane including the surface morphology and height profile taken by a 3D laser scanning microscope. As can be seen in this figure, the membrane was designed and fabricated in a way to have a large pore size (400–500 µm) on one side and a small pore size (50–150 µm) on the other side, to accommodate different cell types at different sides. 

Surgical handling of the 3D-printed membrane was evaluated by two surgeons, and both confirmed its ease of handling, flexibility, and capability of being manipulated in different directions (Figure 2). Moreover, the membrane could lie on the tissue well and cover the defects perfectly. The cutting of the membrane with surgical forceps/scissors, as well as the suturing, were conducted as easily as possible. 

In all evaluated samples, uneventful healing without any sign of inflammation was observed. 

**Figure 4:**
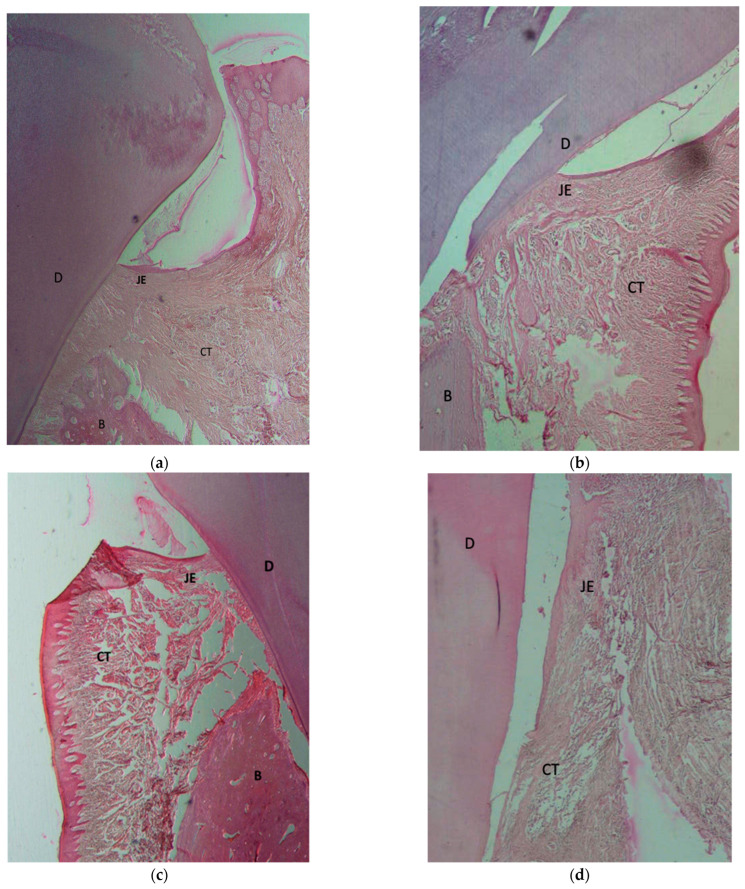
Histomorphometric views of the bucco–lingual section of the critical-size surgically created periodontal defect sites of specimens 8 weeks after interventions. The defect site (5 mm below CEJ) was investigated in all samples. GTR sample treated with (**a**) Botiss—Jason membrane, (**b**) Regedent—Smartbrane membrane, (**c**) 3D-printed membrane. In specimens treated by the GTR technique plus placement of a membrane, there were signs of regeneration of the defect with bone formation, new connective tissue attachment, and formation of a junctional epithelium. (**d**) Control/without membrane. In the control samples, the defect was only filled with long junctional epithelium and CT due to the faster growth rate of the epithelial fibroblasts. (GTR: guided tissue regeneration, B: bone; D: dentin; CT: connective tissue; JE: junctional epithelium).

Histological evaluations (Figure 4) show that in all GTR-treated samples using the three different membranes to cover the defects, new bone formation and regeneration of cementum at the defect sites could be observed. Functionally oriented PDL fibers were also visible between the newly formed cementum and bone, comprising fibroblast cells, collagen fibers, and newly formed blood vessels (Figure 4a). The formation of a healthy junctional epithelium (JE) attachment to the root surface was clearly observed in the coronal portion of the membrane-treated defects (Figure 4a–c).

However, in the control group samples (Figure 4d), only long junctional epithelium and highly vascularized connective tissue were observed on the denuded root surfaces in the defect sites with fibroblast cells and collagen fibers. This was observed without any signs of new attachment formation as seen by new bone formation and creation of a PDL and supra-crestal tissue attachments (JE attachment and insertion of collagen fibers to the root cementum) observed in the GTR-treated samples. Limited bone formation was observed in only three samples of the control group and only in the apical portion of the defects, and there was no obvious sign of cementum formation. 

No sign of root surface resorption was found in any of the samples. 

**Figure 5:**
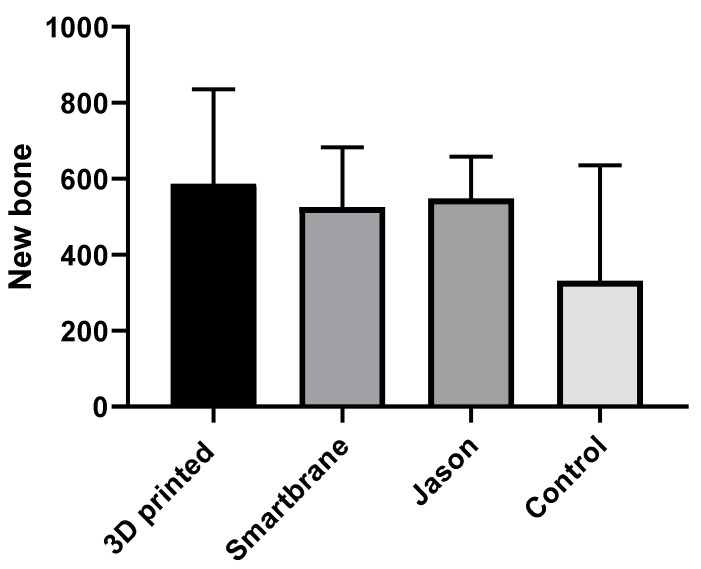
Comparison of maximum new bone thickness (µm) in the four study groups. Mean values and SD.

The results of the histometric evaluation of the defects in each group are shown in Table 1. Histometric analyses indicate an overall higher amount of bone and cementum formation in membrane-treated groups compared to the control group, although these differences were only statistically significant regarding cementum formation (*p* = 0.001). More new bone formation was observed on most of the samples in the group treated with the 3D-printed membrane (Figure 5), although statistically there was no significant difference among the three membrane groups (*p* = 0.594). Cementum formation was also higher in the membrane groups (Smartbrane, Jason, and 3D-printed), compared to the control. Cementum formation results for the 3D-printed membrane and Smartbrane groups were similar (*p* = 0.271). However, the Jason membrane showed statistically significant higher amounts of new cementum formation compared to the 3D-printed (*p* = 0.009) and Smartbrane membranes (*p* = 0.009).

## 4. Discussion

We have selected a 5 mm × 5 mm defect size to follow the requirement for making a critical-size defect. The critical-size defect is a term used in the field of bone repair and regeneration to refer to a defect that is too large to be repaired by the body’s natural healing processes. When a bone is fractured or otherwise damaged, the body’s natural healing process involves the formation of new bone tissue to fill in the defect. However, if the defect is too large, the body may not be able to generate enough new bone tissue to repair it completely. In these cases, the bone defect is considered “critical-size”, and additional intervention may be needed to stimulate healing or repair of the defect. This can include the use of bone grafts, stem cells, or other tissue engineering techniques. The surgically created dehiscence-type critical bone defects in the present study are similar to those previously developed and used by other researchers to study periodontal regeneration [45,46].

The main component of the commercial membranes used in this study was collagen type I/III obtained from the porcine pericardium. The Jason membrane shows a natural honeycomb-like, multilayered collagen structure with increased content of collagen type III, leading to good tear resistance and slow degradation, ensuring a natural long barrier function suitable for the GBR/GTR procedure. Smartbrane is a porcine pericardium-derived membrane with an optimal matrix composition and a naturally dense 3D-network collagen structure. The membranes have an adequate resorption time of about 8–12 weeks as well as a barrier function for GTR treatment. These membranes serve as biocompatible cell barriers due to the presence of a dense fiber network which, at the same time, allows the exchange of fluids and nutrients.

Although there are many reports showing biocompatibility and moderate to low cytotoxicity of collagen [47], in this study we were trying to replace this material with a composition of other hydrogels due to the high cost associated with collagen. In summation, we wanted to show that careful selection of other hydrogels can act as effectively as collagen for GTR application.

The main material that we used as a replacement for collagen was gelatin. Hydrolyzing animal collagen with acid/alkaline can result in gelatin. Thus, gelatin can be considered the denatured form of collagen but is much less expensive. In fact, it is known as one of the most reasonably priced hydrogels for medical and food industry applications, as well as in the pharmaceutical and cosmetic fields [48,49]. “Generally regarded as safe” (GRAS) is how the United States Food and Drug Administration (FDA) describes gelatin [50]. Considering the denatured nature of gelatin, it is antigenic to a lesser extent than collagen. Its mechanical property is also acceptable for GTR application [51]. The presence of some motifs in gelatin, for example, arginine-glycine-aspartic (RGD) sequences, makes it very appropriate for cell attachment. Moreover, its degradation rate makes it practically suitable for GTR application [49,52].

Including a proper amount of elastin and sodium hyaluronate makes the fabricated membrane more appropriate for GTR application. More specifically, elastin can induce long-term stability and elasticity to the membrane [53] and sodium hyaluronate facilitates chemical signaling among cells when they are growing around the membrane [10]. Sodium hyaluronate, also known as hyaluronic acid, is a glycosaminoglycan that is associated with the tissue repair process and plays a prominent role in the wound-healing process. Hyaluronic acid can be found in the extracellular matrix that stimulates cell migration, attachment, differentiation, and proliferation [54,55,56,57].

There are some comprehensive studies on the use of hyaluronic acid as a key material for GTR/GBR or as a carrier of cells in periodontal regenerative and tissue engineering-based therapies [58,59,60,61]. Hyaluronic acid is also known to be osteoconductive, promote angiogenesis, and possess a moderate immune response with additional benefits to be used as an adjunctive to non-surgical and surgical periodontal therapy [60,62,63].

In addition to the advantages of the materials used for the construction of our membrane, the method of its fabrication, i.e., 3D-printing, may allow for the fabrication of membranes specifically designed for GTR application with fitting configuration and size of the defect for wound stability and closure. It has been well documented that wound stability and closure are essential for primary intention healing and the regeneration of periodontal defects [64]. Moreover, 3D-printing techniques have attracted a great deal of attention in periodontal tissue regeneration therapies due to the sophisticated and challenging nature of this kind of reconstruction, which requires the regeneration of three different tissues including bone, cementum, and periodontal ligaments. Studies have recently been conducted focusing on the special design of 3D-printed constructs to promote and guide periodontal regeneration [6,65]. Since each layer of the periodontium has distinctive characteristics, the scaffold for each part can be designed accordingly to achieve multi-tissue regeneration [66]. Formation of a functional connection between cementum/alveolar bone and PDL is of great importance to rebuild the interface between PDL and bone. Three-dimensional printing may offer a solution for making such functional connections.

In the present study, we evaluated periodontal healing on surgically created dehiscence type defects in which periodontal ligaments and cementum were removed manually, and the roots had not been exposed to a periodontal pocket or an oral environment. Studies on the regenerative potential of periodontal tissues have shown no histologic differences in the healing results of periodontitis-affected roots and roots with surgically created defects [67,68,69].

Moreover, previous studies have also shown that after 8 weeks of an ideal GTR, we can expect a complete regeneration. This was evident in all membrane-treated samples obtained in the present study. Histological observation of periodontal healing has previously shown that by 8 weeks, woven bone regeneration can be observed in the coronal aspect of the periodontal defect and lamellar bone is detected in the apical portion. Almost the entire root surface was covered by new cementum with evidence of intrinsic collagen fibers in its ground substance matrix. A mature PDL can be observed in the apical aspect of the defect [70].

We also observed a similar healing and new attachment of the junctional epithelium and collagen fibers of the supracrestal attachment to the newly formed cementum and new bone formation in the defect sites. More fully mineralized bone was found in the apical half of the defects, as well as less mineralized bone in the coronal portions in both collagen membrane groups and 3D-printed membrane samples. The membrane-covered groups all showed successful results for the regeneration of cementum. This cementogenesis is a critical procedure for the new attachment process and the creation of a functioning PDL [71,72,73]. However, healing in control samples only consisted of connective tissue formation in the defect area without the formation of bone and normal supracrestal attachment. This is because membranes can act as a barrier and prevent the rapid apical migration of epithelial into the defect area, allowing cells originating from the periodontal ligament and adjacent alveolar bone to proliferate into the defect site, which results in the regeneration of the periodontium. However, in control cases, the epithelial fibroblasts fill in the defect as they have a faster growth rate compared to precursor cells originating from PDL and bone [74,75,76].

Separation of the gingival epithelium from bone and PDL tissues is the key feature of periodontal GTR barrier membranes. Designing membranes with a proper cell occluding function that can guide activity and attract appropriate cells to the defect area has been the focus in the developments of GTR membranes [13,77]. Behfarnia et al. comparatively evaluated three collagen membranes and reported similar regeneration results of the periodontal tissues at 8 weeks compared to the results observed in the present study [4]. They also reported complete degradation of the membranes at their 4-week evaluation time point. There was also no sign of remaining membranes in any of the groups at the 8-week timepoint in the present study. The results of a study by Akizuki investigating periodontal regeneration after the application of the periodontal ligament cell sheet in critical-size dehiscence defects were also similar to the results observed in the current study [58,60]. They found significantly higher cementum formation, while the difference in bone formation was not statistically significant.

Graziani et al. have reported similar periodontal healing results in their evaluations of GTR treatment with resorbable PLA (Guidore membrane) and non-resorbable ePTFE (Gortex) in dehiscence-type defects in monkeys. They observed that about a 10–20 µm thin layer of acellular extrinsic fiber cementum (AEFC) had covered the instrumented root surface after 6 weeks, which was similar to the results observed in the membrane groups of our current study. They reported that the maturation process continued over at least 2 years in GTR-treated defect sites [70].

In another similar study, Polimeni et al. evaluated the regeneration of dehiscence- type periodontal defects using ePTFE reinforced GORE-TEX non-resorbable membranes in dogs of the Beagle breed [64]. Although similar to the current study, they have also reported higher regeneration of cemental tissues compared to bone regeneration, and they have stated that different periodontal tissues’ formation occurred in parallel with each other, and space provided by the membrane played an important role in the regeneration of these tissues.

To summarize the results, periodontal tissue regeneration was observed in all membrane-treated groups (groups 1–3) and all membranes degraded without any signs of inflammation within 8 weeks after surgery. However, in the control group, connective tissue was formed on root surfaces in defect sites. Histometric analysis showed that the formation of cementum in the experimental groups was significantly higher than that in the control group (*p* = 0.001). However, statistically more new cementum formation was observed in sites treated with the Jason membrane when compared to the 3D-printed membrane and Smartbrane. New bone formation was also higher in the group treated by the 3D-printed membrane (Figure 5). However, the difference between the three experimental membrane groups (Jason, Smartbrane, and 3D-printed membrane) was not statistically significant (*p* = 0.594). Generally, the defects treated by the designed 3D-printed membrane revealed comparable outcomes to the defects treated by commercially available collagen membranes. The issues related to observation with no statistically significant results, which is very common in animal studies, are usually related to the short follow-up of the study and the low number of samples in each group, which is practically difficult to be increased due to the use of large animals. Thus, further studies using a greater number of animals and longer follow-up studies evaluating the maturation of the defects treated with each type of membrane are recommended.

## 5. Conclusions

The results showed that the 3D-printed membrane had completely degraded without any side effects. Based on the histological data, the group treated with the 3D-printed membrane presented more new bone formation compared to the other groups. In general, the function of the 3D-printed membrane was observed to be better or comparable with commercial membranes.

Since Jason and Smartbrane membranes are made of collagen, we may conclude that the composition of our designed 3D-printed soft membrane can be an effective and affordable replacement for collagen which is an expensive material to be used in GTR membranes.

## Figures and Tables

**Table 1:** Histomorphometric measurements of newly formed tissues.

		N	Mean	Std Deviation	Std Error	95% Confidence Interval for Mean		Minimum	Maximum
						Lower Bound	Upper Bound		
New Bone (µm)	3D-printed	5	586.68	248.7309	111.2358	277.8399	895.5201	232	874.6
	Smartbrane	4	525.825	156.8492	78.4246	276.2429	775.4071	332.1	700.2
	Jason	4	547.575	110.5278	55.26392	371.7005	723.4495	427.4	680.3
	Control	5	331.34	303.8702	135.8949	−45.9646	708.6446	0	599.8
	Total	18	493.5389	232.3839	54.77341	377.9771	609.1007	0	874.6
New Cementum (µm)	3D printed	5	7.7	1.23491	0.55227	6.1667	9.2333	6.3	9.1
	Smartbrane	6	11.5	5.61534	2.29245	5.6071	17.3929	6	22
	Jason	5	15.7	2.80268	1.2534	12.22	19.18	13.5	20
	Control	5	0	0	0	0	0	0	0
	Total	21	8.8571	6.59603	1.43937	5.8547	11.8596	0	22
